# Consequences of Job Insecurity on the Psychological and Physical Health of Greek Civil Servants

**DOI:** 10.1155/2015/673623

**Published:** 2015-10-18

**Authors:** Dimitra Nella, Efharis Panagopoulou, Nikiforos Galanis, Anthony Montgomery, Alexis Benos

**Affiliations:** ^1^School of Medicine, Faculty of Health Sciences, Aristotle University of Thessaloniki, University Campus, 55131 Thessaloniki, Greece; ^2^Department of Orthopaedics, “Papageorgiou” General Hospital, Medical School, Aristotle University of Thessaloniki, 56403 Thessaloniki, Greece; ^3^Department of Educational and Social Policy, University of Macedonia, Egnatia Street 156, 54636 Thessaloniki, Greece

## Abstract

The aim of this study was to estimate the short term consequences of job insecurity associated with a newly introduced mobility framework in Greece. In specific, the study examined the impact of job insecurity on anxiety, depression, and psychosomatic and musculoskeletal symptoms, two months after the announcement of the mobility framework. In addition the study also examined the “spill over” effects of job insecurity on employees not directly affected by the mobility framework. Personal interviews using a structured questionnaire were conducted for 36 university administrative employees awaiting repositioning, 36 coworkers not at risk, and 28 administrative employees of a local hospital not at risk. Compared to both control groups the employees in the anticipation phase of labor mobility had significantly worse scores for perceived stress, anxiety, depression, positive affect, negative affect, social support, marital discord, common somatic symptoms, and frequency of musculoskeletal pain. This study highlights the immediate detrimental effects of job insecurity on the physical, psychological, and social functioning of employees. There is a need for the development of front line interventions to prevent these effects from developing into chronic conditions with considerable cost for the individual and society in general.

## 1. Introduction

Since 2008 and the beginning of the Economic Crisis that has affected most European countries including Greece, new forms of flexible and marginal employment have emerged resulting in a considerable increase in job insecurity. Job insecurity is a social phenomenon, meaning that it is experienced as a subjective perception about employment and unemployment, and reflects the insecurity, uncertainty, powerlessness and helplessness that occur when an individuals lacks the assurances that their job will remain stable [1]. It has been stated that job insecurity is the most stressful aspect of the process leading to unemployment with a worse impact on employees than unemployment itself [2].

Job insecurity has been defined as the subjectively perceived and undesired possibility to lose the present job in the future, as well as the fear or worries related to the possibility of job loss [1, 3]. It can be differentiated between cognitive and affective job insecurity with the first referring to the cognitive probability of losing one's job and the latter referring to the fear and worry of losing one's work. Another way to differentiate job insecurity is differentiating between quantitative insecurity which refers to worrying about the loss of job itself and qualitative which refers to worrying about losing important aspects of job, for example, salary, health insurance, and social life [1, 3, 4].

Job insecurity varies by race, ethnicity, and immigration status [5]. In two nationally representative US samples, more Blacks than non-Blacks experienced perceived job insecurity [6]. Immigrant women in Sweden were more likely to work in temporary jobs than native born women [7]. Erlinghagen [8] analyzed self-perceived job insecurity among 17 European countries and found significant cross-country differences in individuals' perception of job insecurity. Not only were the findings driven by social-structural or institutional differences, but also the perception of job insecurity was influenced by nation-specific unobserved characteristics (e.g., religiousness, general assessment of job security, and basic trust in fellow human beings).

Risk factors for experiencing job insecurity include being male, a finding linked to the traditional role of the breadwinner, age between 30–44 years old, lower educational status, being self-employed or working in the private practice, blue collar work, and working in the manufacturing sector [9, 10].

Job insecurity has been linked to several adverse health outcomes. In regard to mental health it has been associated with psychosomatic symptoms, loss of self-esteem, anxiety, and minor psychiatric symptoms [1, 11–14]. In regard to physical health, job insecurity has been associated with increased morbidity, lower levels of self-reported health, increased incidence rates of hypertension, coronary heart disease, and myocardial death [15, 16]. In addition, job insecurity has been found to lead to restricted physical activity due to musculoskeletal disorders such as low back pain and neck pain [17, 18]. In addition, anticipation of redundancy has been shown to affect health behaviors such as exercise, dietary habits, and sleep [19–21]. Moreover, job insecurity is associated with increased use of healthcare services and decreased compliance with occupational safety regulations [22].

Three theoretical models have been suggested to explain the paths that lead from the negative consequences of job insecurity to employees' health and wellbeing, Jahoda's latent deprivation model [23], the psychological contract theory [24, 25], and the vitamin model suggested by Probst and Brubaker [22]. Jahoda's model suggests that the possibility of losing one's job threatens the satisfaction of needs such as income and social contacts and leads to frustration. The second model, concerning psychological contract theory, suggests that insecurity about retaining their posts is perceived by employees as a violation of an untold contract on behalf of their employer and therefore their commitment to their business and their well-being is affected. Finally, according to the vitamin model, job insecurity affects negatively employees' wellbeing due to the associated feelings of unpredictability and uncontrollability [26].

In 2012, the Greek government launched a reform of the national civil service, as part of dealing with the ongoing financial crisis. The published law 4093/2012 highlighted the need for the suspension of almost 2000 civil servants throughout the Greek public sector through a suggested “mobility framework for personnel.” The framework dictated that a percentage of employees of the civil service, following an assessment process, would be transferred to other posts within the country with 75% of their current salary. Following the transfer, and a second evaluation process, a percentage of those employees would eventually be made redundant from the civil service.

The reform programme in Greece has resulted in heightened feelings of job instability and job insecurity. A review of the literature indicates that job instability/insecurity is associated with psychologically ill health and impaired physical health [5]. Indeed, chronic job insecurity appears to have a dose-response relationship with self-reported health and physical symptoms and increases the risk of minor psychiatric morbidity [27, 28]. Greece has been profoundly affected by the global financial and economic crisis, with wide-ranging economic, social, and political consequences. In terms of job insecurity, the picture in Greece is bleak. In 2015, the country is entering its seventh year of recession and is still operating within severely constricted fiscal limits. Public and nonprofit mental health service providers have scaled back operations, shut down, or reduced staff; plans for the development of child psychiatric services have been abandoned; and state funding for mental health decreased by 20% between 2010 and 2011, and by a further 55% between 2011 and 2012 [29]. There has been a substantial deterioration in mental health status, with population surveys suggesting a significant increase in the prevalence of major depression, from 3.3% in 2008 to 8.2% in 2011, with economic hardship being a major risk factor [30].

The aim of this study was to estimate the short term consequences of job insecurity associated with the newly introduced mobility framework. In specific, the study examined the impact of job insecurity on anxiety, depression, psychosomatic and musculoskeletal symptoms, two months after the announcement of the mobility framework. In addition the study also examined the “spill over” effects of job insecurity in employees not directly affected by the mobility framework.

## 2. Materials and Methods

Employees of the administration department of the Aristotle University of Thessaloniki, expecting to be made redundant after the publication of the mobility framework, were recruited for the study. In order to be included in the study employees had to be still working in their current post. The comparison group consisted of employees of the administration department of an academic hospital not at risk of job redundancy at the time of the study. To examine the “spill over” effects of job insecurity, a second comparison group was created, consisting of employees of the administration department of the same University, not at risk of losing their jobs.

The study was approved by the Ethical board of the Medical School of the Aristotle University of Thessaloniki. All employees were informed about the study with an email inviting them to participate and explaining the purpose of the study. A telephone contact number was given for employees interested in participating. After the first telephone contact with the research team, an appointment was set for the interview. Interviews took place during working hours in a private room in the employees' working place. All interviews were conducted by the same researcher. At the beginning of the interview participants were ensured about the anonymity of the procedure and gave their verbal consent. At the end of the interview all respondents were given contact information for counseling and support groups. Participants were informed of their right to withdraw from the study at any point. Collected data were stored appropriately in a secure location.

The present study is a prospective study that investigates a change in job security in a sample of employees, who were compared with a suitable cohort of employees who did not experience a change in job security. The employed methodology is consistent with researchers who emphasize the importance of exploring job insecurity via “natural experiments” [31, 32].

The questionnaire consisted of nine parts. For parts 1 to 7 the Greek versions of the Perceived Stress Scale (PSS-10) [33, 34], Hospital Anxiety and Depression Scale (HADS) [35–37], Multidimensional Scale of Perceived Social Support (MSPSS) [38, 39], the Beier-Sternberg Discord Questionnaire (DQ) [40], the Positive and Negative Affect Schedules (PANAS) [41], the Pennebaker Inventory of Limbic Languidness (PILL) [42], and the Health Behavior Inventory (HBI) [43, 44] were used to assess perceived stress, anxiety and depression, social support, marital discord, positive and negative affect, common somatic symptoms, and health behaviors, respectively. Part 8 consisted of a body diagram with all main joints to estimate the frequency of musculoskeletal pain in a scale of 1 to 10 based on the Nordic questionnaire [45] (MS scale) and part 9 consisted of demographic and medical history information.

Statistical analysis was performed using the statistical package IBM SPSS Statistics Standard v.20 and statistical significance was set up at *p* < 0.05. Patient demographics were summarized using descriptive statistics. Scores for all scales used were calculated. For the HADS questionnaire, the cutoff score of 8 was applied suggested by the authors to identify clinical cases of anxiety disorder and depression. Due to the fact that none of the scales were normally distributed, nonparametric statistics were employed. Median and interquartile range were chosen as descriptive measures. Kruskal Wallis tests were used to compare all study groups. In case of significant differences, Mann-Whitney *U* tests were used to compare scores between two groups.

## 3. Results

The final sample consisted of 36 out of 97 (37% response rate) of university employees at risk of being made redundant, 36 out of 114 (31.5% response rate) of university administrative employees not at present risk of labor mobility, and 28 out of 105 hospital administrative employees (26% response rate).

Demographic characteristics of the sample are summarized in Table 1.

Results showed that, with the exception of marital discord (DQ), scores for all other aspects of psychosocial and physical health were significantly different between all three groups (Table 2).

Further comparisons using Mann-Whitney *U* test indicated that the “job insecurity” group had higher scores for perceived stress (*p* < 0.001), anxiety (*p* < 0.001), depression (*p* < 0.001), negative affect (*p* < 0.001), common somatic symptoms (*p* < 0.001), and musculoskeletal pain (*p* < 0.001) and lower scores for positive affect (*p* < 0.001) and perceived social support (*p* = 0.011 and *p* = 0.006) compared to each one of the control groups, while no difference was shown between the two control groups (Table 3).

In terms of clinical cases of depression and anxiety, 35 (97%) of participants in the job insecurity group were classified as anxiety disorder cases compared to the 16 (25%) of participants in each one of the control groups (*χ* ^2^ = 51.9, *p* < 0.001). This corresponds to an OR = 105 probabilities (95% CI 13.3–829.4, *p* < 0.001) for office workers in the job insecurity group to develop an anxiety disorder. Likewise for subscale HADS-D for depression, 31 (86%) of participants in the job insecurity group were defined as cases for depression against 2 (3%) participants in both control groups (*χ* ^2^ = 71.8, *p* < 0.001 Monte Carlo method), corresponding to a probability of OR = 192.2 (95% CI 35.3–1047.4, *p* < 0.001) for participants in the job insecurity group to develop depression.

Protective and high risk health behaviors and health care use were assessed and compared between the investigation group and the two groups together (Table 4). Office workers in the job insecurity group reported eating more fast food meals per week than workers not at risk (*p* = 0.001). They also consumed more coffees (*p* = 0.001) and cigarettes per day (*p* = 0.001). In addition they engaged in less physical exercise per week (*p* = 0.001), slept less during the night (*p* < 0.001), and used more medication for pain relief (*p* < 0.001). Finally, employees feeling insecure about their job visited the doctor more often during the past month (*p* = 0.048) and reported more absent days due to sickness (*p* = 0.034) compared to the comparison groups.

## 4. Discussion

Results show that employees at risk of losing their jobs showed higher levels of perceived stress, anxiety, depression, and negative feelings and lower levels of positive feelings compared to employees not at risk of losing their jobs. These findings are in agreement with previous studies [11, 31]. Results also showed that 97% and 86% of the group at risk of joblessness were classified as clinical cases of anxiety and depression, respectively. As this study was carried out within the first three months of the announcement of the labor shortage measures, results of the study reflect the immediate, short term reaction. However, our results need to be treated with caution as we are unaware of the long term impact of the economic crisis in Greece [46] and its potential impact on our sample.

Employees in the job insecurity group also reported receiving less social support. Additionally, while the result was not statistically significant the job insecurity group did report a higher degree of discord with their spouses. Jenkins et al. have also reported the negative impact of job insecurity on employees' marriages resulting in higher rate of divorces [47]. However, the present data indicates that the relationship between job insecurity and health problems in not related to marital discord.

Employees in the job insecurity group reported higher frequency of common somatic symptoms. Among the symptoms that were most frequently reported were chest pains, racing heart, and choking sensation, symptoms indicative of cardiovascular impairment. Previous studies have also explored the link between job insecurity and cardiovascular symptoms [14] and it is reported that feeling insecure about retaining one's job is a risk factor for coronary heart disease [48]. Both control groups showed significantly lower frequency of musculoskeletal pain compared to employees in anticipation of job loss in accordance with studies reporting that job insecurity increases the risk for low back pain [16] and is a predictor for musculoskeletal pain in the limbs [49].

In terms of health behaviors participants in the job insecurity group smoked more cigarettes per day, exercised less, slept fewer hours every night (under 6 hours per night), and used more frequently painkillers, a finding which can be linked to the increased reported frequency of musculoskeletal pain in the job insecurity group. These results are in agreement with previous studies showing that job insecurity was a risk factor for work-related sleep problems [18] and that more insecure employees tend to drink alcohol, smoke, and not exercise [19, 20, 50].

The reported increased use of health resources by participants in the job insecurity group is in contrast to previous studies showing that the economic crisis in Greece has resulted in a reduction in visits to the doctor [46].

Results showed no spillover effects of the negative consequences of job insecurity on employees working in proximity but not currently at risk of losing their job. This could be time-related as secondary effects of insecurity might need more time to develop. For example in the study of Lang et al. musculoskeletal problems of employees that survived a downsizing of staff were stronger relative to musculoskeletal sickness absences measured for an extended period covering two subsequent years after downsizing [17], suggesting that negative effects may be more pronounced later on.

One of the main study limitations is that no subjective measure of job insecurity was used to assess job insecurity. Employees whose names were included in the first mobility scheme which was introduced by the Greek Government were considered as experiencing job insecurity. Our job insecurity group, individuals on the mobility scheme, were self-selected but we were unable to control for the long term impact of the crisis our participants [46]. This methodology has been previously used in other studies assessing the effects of attributed job insecurity [19, 20, 51]. Future studies should also include subjective assessments of insecurity in order to potentially identify risk and protective factors that can aggravate or alleviate the threat of an objective situation of anticipated job loss. In addition the small, nonrandomised sample size does not permit generalizability of findings to other sectors. Finally, the cross-sectional nature of the study and the fact that it relied on self-report limit our ability to be conclusive. However, the data collected on musculoskeletal pain, demographics, and medical information used a reliable measure that reduced the effect of self-report.

## 5. Conclusion

This study highlights the immediate detrimental effects of job insecurity on physical, psychological, and social functioning of employees. Results of the study also highlight the need for development of front line interventions to prevent these effects from developing into chronic conditions with considerable cost for the individual and society in general. In terms of developing interventions, education appears to be a critical factor. For example, Perlman and Bobak [52] assessed the contribution of unstable employment to mortality in Posttransition Russia and found that unemployment and job insecurity were significant predictors of mortality. One aspect of the study is particularly noteworthy with regard to the importance of education. Perlman and Bobak found that education seemed to provide a protective factor against some indicators of unstable employment, independently of occupation. Although the reasons are uncertain, it is possible that education may provide resilience or coping skills, as suggested by the association between education and higher perceived control [53] or depressive symptoms [54].

## Figures and Tables

**Table 1:** Sample characteristics.

	University office workers in labor mobility	University office workers not at risk	Hospital office workers not at risk	*p* ^*∗*^
	*N* = 36	*N* = 36	*N* = 36	
Gender				
Male	12 (33%)	13 (36%)	9 (32%)	NS
Female	24 (67%)	23 (64%)	19 (68%)	
Age	43.7 ± 7.5^†^	41.6 ± 7.4^†^	41.5 ± 7.9^†^	NS^*∗∗*^
Marital status				
Not married/not in relationship	8 (22%)	9 (25%)	6 (21%)	NS
Married/in relationship	28 (78%)	27 (75%)	22 (79%)	
Education				
Secondary	23 (64%)	9 (25%)	9 (32%)	0.001
Postsecondary	11 (31%)	15 (42%)	7 (25%)	
tertiary	2 (5%)	12 (33%)	12 (43%)	
Employment contract				
Fixed term	0	2 (6%)	1 (4%)	0.03
Not fixed term	36 (100%)	27 (75%)	24 (85%)	
Permanent	0	7 (19%)	3 (11%)	

^*∗*^ *χ* ^2^ test (with Monte Carlo method when needed).

^*∗∗*^ANOVA.

^†^Mean ± SD.

NS: nonsignificant.

**Table 2:** Scores for all scales used concerning psychological and physical health and social support.

	Investigation group	Control group 1	Control group 2	*p* ^*∗*^
	University office workers in labor mobility	University office workers not at risk	Hospital office workers not at risk	
	*N* = 36	*N* = 36	*N* = 36	
	Median (interquartile range)		
PSS-10	27.0 (4.5)	8.5 (5.75)	6.5 (11.5)	<0.001
HADSA	16.0 (3.75)	4.0 (4.5)	3.5 (5.25)	<0.001
HADSD	10.0 (4.0)	2.0 (1.75)	2.0 (3.0)	<0.001
PANAS PA	28.0 (7.5)	41.0 (5.75)	44.0 (6.25)	<0.001
PANAS NA	27.0 (8.75)	15.5 (3.0)	17.0 (5.0)	<0.001
MSPSS	5.79 (1.15)	6.33 (1.02)	6.42 (1.29)	0.008
DQ^*∗∗*^	2.7 (2.1)	2.1 (1.05)	2.45 (1.0)	NS
PILL	4.23 (2.52)	1.1 (0.39)	1.15 (0.5)	<0.001
MS	2.67 (2.13)	1.08 (1.0)	1.0 (0.67)	<0.001

^*∗*^Kruskal Wallis test.

^*∗∗*^For scale DQ group sizes are 27, 26, and 22, respectively.

NS: nonsignificant.

**Table 3:** Comparisons between groups for all scales investigated.

	Investigation group versus control group 1	Investigation group versus control group 2	Control group 1 versus control group 2	Investigation group versus control group 1 + 2
PSS-10	*U* = 35.0	*U* = 30.5	*U* = 463.5	*U* = 65.5
	*p* < 0.001	*p* < 0.001	*p* = NS	*p* < 0.001

HADSA	*U* = 25.5	*U* = 31.0	*U* = 487.5	*U* = 56.5
	*p* < 0.001	*p* < 0.001	*p* = NS	*p* < 0.001

HADSD	*U* = 45.0	*U* = 32.0	*U* = 486.0	*U* = 77.0
	*p* < 0.001	*p* < 0.001	*p* = NS	*p* < 0.001

PANAS PA	*U* = 136.0	*U* = 110.5	*U* = 356.0	*U* = 246.5
	*p* < 0.001	*p* < 0.001	*p* = NS	*p* < 0.001

PANAS NA	*U* = 62.5	*U* = 71.5	*U* = 406.0	*U* = 134.0
	*p* < 0.001	*p* < 0.001	*p* = NS	*p* < 0.001

MSPSS	*U* = 423.0	*U* = 303.0	*U* = 454.5	*U* = 726.0
	*p* = 0.011	*p* = 0.006	*p* = NS	*p* = 0.002

DQ	*U* = 242.0	*U* = 228.5	*U* = 246.5	*U* = 470.5
	*p* = NS	*p* = NS	*p* = NS	*p* = 0.05

PILL	*U* = 64.5	*U* = 38.0	*U* = 483.5	*U* = 102.5
	*p* < 0.001	*p* < 0.001	*p* = NS	*p* < 0.001

MS	*U* = 238.5	*U* = 182.0	*U* = 486.0	*U* = 420.5
	*p* < 0.001	*p* < 0.001	*p* = NS	*p* < 0.001

*U* = Mann-Whitney *U* test.

NS: nonsignificant.

**Table 4:** Health behavior and health care service use.

	Investigation group	Control group 1 + 2	*p* ^*∗*^
	*N* = 36	*N* = 64	
	Median (interquartile range)		
*Protective health behaviors *			
Breakfasts per week	3.5 (6.75)	5.0 (4.0)	NS
Regular meals per week	2.0 (2.0)	2.0 (1.0)	NS
Hours of night sleep per day	6.0 (2.0)	7.0 (1.0)	<0.001
Workout sessions per week	0.0 (0.0)	1.5 (3.0)	0.001

*High risk health behaviors *			
Snacks per week	4.0 (4.0)	2.0 (3.75)	0.001
Cigarettes smokes per day	10.0 (20.0)	0.0 (10.0)	0.001
Coffees per day	2.0 (2.0)	1.5 (1.0)	0.001
Frequency of alcohol drinking per week	1.0 (2.0)	0.0 (1.0)	NS
Alcoholic drinks per week	0.5 (1.75)	0.0 (1.0)	NS
Sleeping pills per week	0.3 ± 1.2^*∗∗*^	0.1 ± 0.9^*∗∗*^	NS
Tranquilizers per week	0.3 ± 1.2^*∗∗*^	0.1 ± 0.9^*∗∗*^	NS
Nonpharmaceutical methods to achieve			
sleep per week	1.0 ± 1.8^*∗∗*^	0.4 ± 1.4^*∗∗*^	0.015
Painkillers per week	1.8 ± 2.1^*∗∗*^	0.3 ± 1.0^*∗∗*^	<0.001

*Health care services use *			
Medical consultations per month	0.4 ± 0.8^*∗∗*^	0.2 ± 0.6^*∗∗*^	0.048
Sick days per month	0.3 ± 0.9^*∗∗*^	0.0 ± 0.1^*∗∗*^	0.034

^*∗*^Mann-Whitney *U* test.

^*∗∗*^Median ± SD.

NS: nonsignificant.
